# A Bioinspired Ag Nanoparticle/PPy Nanobowl/TiO_2_ Micropyramid SERS Substrate

**DOI:** 10.3390/nano12224104

**Published:** 2022-11-21

**Authors:** Xin Li, Florian Ion Tiberiu Petrescu, Qupei Danzeng, Haiyan Zhu, Ying Li, Gang Shi

**Affiliations:** 1 Key Laboratory of Synthetic and Biotechnology Colloids, Ministry of Education; School of Chemical and Material Engineering, Jiangnan University, Wuxi 214122, China; 2 Department of Mechanisms and Robots Theory, Bucharest Polytechnic University, 060042 Bucharest, Romania; 3 Department of Tibetan medicine; University of Tibetan Medicine, Lhasa 540100, China

**Keywords:** TiO_2_ micropyramid, PPy nanobowl, SERS, soft imprinting, bioinspired

## Abstract

In this paper, the micropyramid structure was transferred to the TiO_2_ substrate by soft imprinting. Then, the PPy nanobowls were assembled onto the surface of the TiO_2_ micropyramids through the induction of the PS template. Finally, a layer of Ag nanoparticles was deposited on the surface of PPy nanobowls to form a novel Ag nanoparticle/PPy nanobowl/TiO_2_ micropyramid SERS substrate. Its structure is similar to the bioinspired compound eyes. This substrate exhibited excellent antireflection, ultra-sensitivity, excellent uniformity, and recyclability. The concentration of R6G molecules can be detected as low as 10^−9^ mol/L, and the Raman enhancement factor can reach 3.4 × 10^5^. In addition, the excellent catalytic degradation performance of the substrate ensures recyclability. This work proves that the micropyramid structure can be applied to other SERS materials besides silicon by the above methods, which broadens the selection range of composite SERS materials.

## 1. Introduction

Composites with a specific structure can not only retain the intrinsic properties of each component, but also can impart other special functions to the composites [1]. Structural regulation of composites in the development of new functional materials has excellent application prospects, such as effective utilization of energy, pollution treatment, and functional detection [2,3,4]. Periodical-ordered bioinspired structures play an important role in the fabrication of composites, which can be applied to photonic crystals, flat panel displays, and antireflection coatings, because of the effective improvement in the mechanical, optical, and electrical properties [5,6,7]. In particular, some bioinspired structures combined with silicon (Si) micropyramids are of interest, which can efficiently absorb incident light. According to the equivalent medium theory [8,9], the micropyramid structure fabricated on the monocrystalline Si surface shows an excellent antireflection effect due to the gradual change in the refractive index from air to Si [10,11]. In addition, the micropyramid structure has great potential in microelectronic devices such as transistors, semiconductor chips, and sensors [12,13,14]. In order to apply this kind of nanostructure to other materials, many nanomechanical technologies have been developed, such as soft imprinting, self-assembly, nanoimprinting, electron beam etching, and so on [15,16,17,18]. However, most of the current works are mainly focused on the fabrication of pyramid Si, which limited the application field.

In our previous works [19,20], the Si-based micropyramid structure was applied to the fabrication of the SERS substrate, which showed excellent anti-reflection and high detection sensitivity. TiO_2_ is an excellent photocatalytic material. If the TiO_2_ micropyramid structure can be fabricated and applied to the SERS substrate, its detection sensitivity will be improved through the anti-reflective performance of TiO_2_ micropyramids, and its reuse will be realized through the photocatalytic performance of TiO_2_. Here, the TiO_2_ pyramids were first fabricated by soft imprinting. Then, the polypyrrole (Ppy) nanobowls were assembled onto the surface of the TiO_2_ micropyramids. The uniformly distributed Ag nanoparticles were subsequently modified on the surface of PPy nanobowls, to form a novel SERS substrate, which structure is similar to the bioinspired compound eyes. This structure exhibited excellent anti-reflective performance and the nanobowl structure was conducive to the enrichment of probe molecules to improve the Raman detection limit of the SERS substrate. At the same time, the focusing effect of the nanoscale bowl structure is conducive to improving the plasma resonance of Ag nanoparticles, thereby improving the Raman signal. Furthermore, the antireflection, photoelectric, photocatalytic, and SERS performance of the composite substrate were also investigated.

## 2. Materials and Methods

### 2.1. Chemicals and Materials

Acetone (CH_3_COCH_3_), chloroform (CHCl_3_), ethanol (CH_3_CH_2_OH), ammonium hydroxide (NH_3_·H_2_O), hydrogen peroxide (H_2_O_2_), hydrochloric acid (HCl), potassium hydroxide (KOH), n-butyl titanate (C_16_H_36_O_4_Ti), pyrrole (Py, C_4_H_5_N), stannous chloride dihydrate (SnCl_2_·2H_2_O), sodium dodecyl sulfate (SDS), rhodamine 6G (R6G), and potassium persulfate (KPS) were purchased from Sinopharm Chemical Reagent Co., LTD., Shanghai, China. Silver nitrate (AgNO_3_), polydimethylsiloxane (PDMS, (C_2_H_6_OSi)_n_), and styrene (C_8_H_8_) were purchased from Sigma Aldrich Trading Co., LTD., St. Louis, MO, USA. All reagents were used directly without further purification. The Si wafers (p-type (100)) were obtained from Youyan Guigu, Beijing, China.

### 2.2. Fabrication of PDMS Template

Firstly, the Si wafer with 1 cm × 2 cm was washed with acetone, chloroform, ethanol, and deionized water successively to remove the impurities. Then, the hydrophilic treatment of Si wafer was placed in a solution (the volume ratio of NH_3_.H_2_O: H_2_O_2_: H_2_O was 1:1:5) at 80 °C. Subsequently, the Si wafer was etched to form the pyramid Si (p-Si) in a KOH solution for 35 min at 90 °C [21,22,23]. Whereafter, the PDMS prepolymer with the curing agent in a mass ratio of 10: 1 was poured onto the silicon micro-pyramids. After curing for 3 h at 75 °C, the inverted pyramid PDMS template was obtained [24,25].

### 2.3. Fabrication of Pyramid TiO_2_

TiO_2_ sol was synthesized with the typical sol–gel method [26]. Then, 60 μL of TiO_2_ sol was dropped on the Si wafer (2 cm × 2 cm) and imprinted with the PDMS template immediately. The above sample was placed at room temperature for 24 h until the solvent volatilized completely. The pyramid TiO_2_ (p-TiO_2_) was obtained after the PDMS template, and the sample was separated. Finally, the pyramid TiO_2_ was calcined in a tube furnace at a heating rate of 1 °C/min to 450 °C for 3 h.

### 2.4. Assembly of PPy Nanobowls and Ag Nanoparticles on p-TiO_2_

The 500 nm polystyrene (PS) microsphere prepared in our laboratory was dropped onto the Py solution with SDS to form a monolayer of closely packed PS spheres [20]. Then, FeCl_3_ was added to the above solution as an initiator to polymerize Py into PPy, which experimental details refer to our previous work [27]. After 30 min of polymerization, the closely packed single-layer PS spheres with PPy were transferred to the surface of p-TiO_2_. After drying at room temperature, the above sample was treated with toluene to remove the PS spheres, and then p-TiO_2_ with PPy nanobowls (b-PPy/p-TiO_2_) on the surface of Si was obtained. Finally, Ag nanoparticles were deposited onto the surface of b-PPy/p-TiO_2_ to form Ag/b-PPy/p-TiO_2_ by the typical reduction method [20].

### 2.5. Characterization

The different samples were pasted onto the conductive adhesive, and the microstructure of them was tested by field emission scanning electron microscopy (SEM, S-4800, Hitachi, Tokyo, Japan). UV–visible near-infrared spectrophotometer (UV-3600plus, Shimazu Company, Kyoto, Japan) was used to analyze the reflectance of the samples. X-ray powder diffraction (XRD, Bruker AXS D8, Karlsruhe, Germany) was used to test the crystal patterns of the samples. The photoelectric properties of the samples were analyzed by the electrochemistry station (CHI660, Shanghai Chenhua Company, Shanghai, China), in which the sample, the conductive glass, and Ag/AgCl was used as the working electrode, the counter electrode, and the reference electrode, respectively, and 0.5 mol/L of Na_2_SO_4_ as the electrolyte. The samples were illuminated by the simulated solar through a Pt sheet to test the photoelectric performance. R6G was photodegraded under visible light irradiation. The absorption spectrum of the solution was recorded with a UV–visible spectrophotometer (TU-1901, Beijing Persee General Instrument Co., LTD., Beijing, China) to analyze the photocatalytic performance of the SERS substrate. The Raman performance of the SERS substrate was detected by a confocal microscopy Raman spectrometer (Renishaw inVia, Renishaw PLC, Gloucestershire, UK), and R6G was used as the probe molecule.

## 3. Results and Discussion

### 3.1. Fabrication of Ag/b-PPy/p-TiO_2_ SERS Substrate

**Scheme 1:**
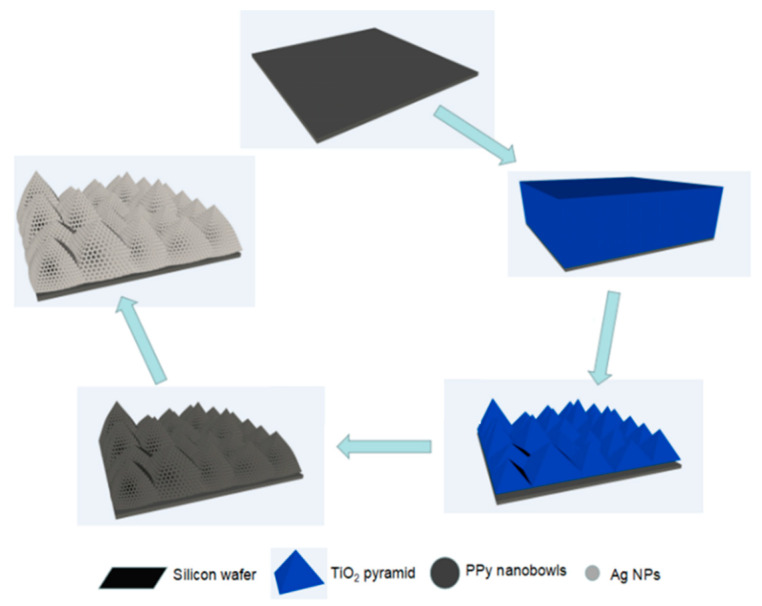
Scheme of fabricating the Ag/b-PPy/p-TiO_2_ SERS substrate.

**Figure 1:**
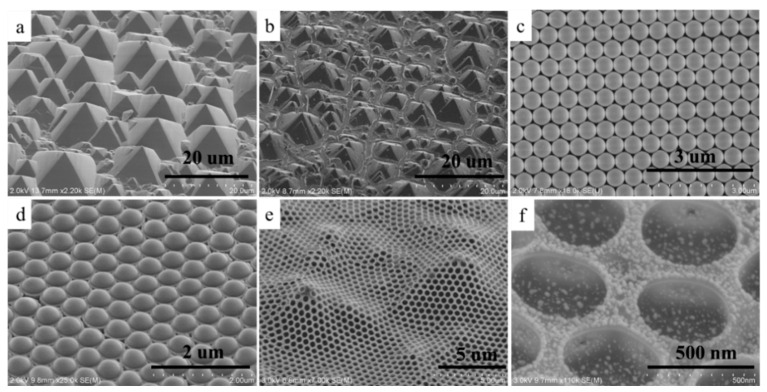
SEM photographs of (**a**) p-Si pyramids, (**b**) p-TiO_2_, (**c**) self-assembled PS microspheres, (**d**) PS/PPy film fabricated from PPy solution, (**e**) b-PPy/p-TiO_2_, (**f**) Ag/b-PPy/p-TiO_2_.

**Figure 2:**
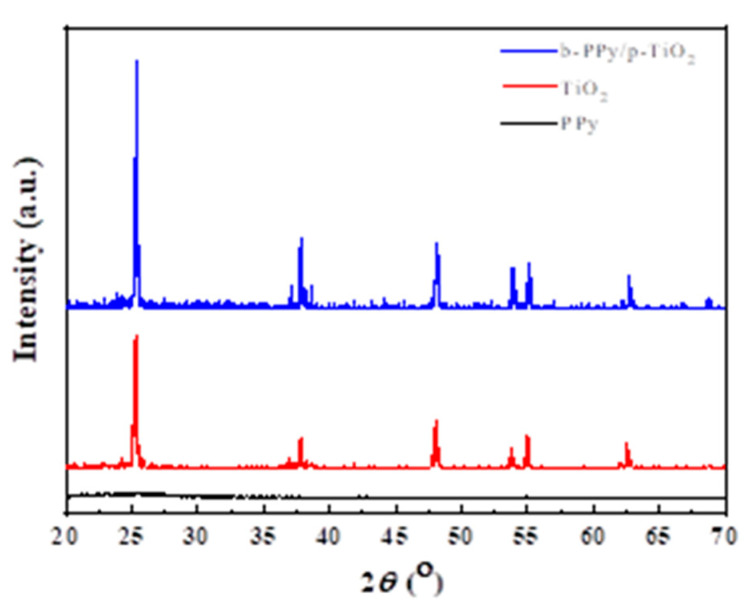
XRD patterns of PPy, TiO_2_ and b-PPy/p-TiO_2_.

The fabrication process of the Ag/b-PPy/p-TiO_2_ SERS substrate is shown in Scheme 1. Firstly, the Si micropyramids (p-Si) were obtained by anisotropic etching of alkali. It can be seen from Figure 1a that the closely arranged micropyramids is all over the Si surface. After the PDMS template was duplicated from the p-Si, the TiO_2_ micropyramids (p-TiO_2_) were fabricated through the soft imprinting method. Subsequently, the p-TiO_2_ substrate (Figure 1b) was calcinated, which morphology and XRD pattern are shown in Figure 1b and Figure 2, indicating that the p-TiO_2_ morphology is similar to that of p-Si and TiO_2_ is anatase type. Meanwhile, the PS spheres, which morphology is shown in Figure 1c, were transferred to the Py solution. After a period of time, a certain amount of Py was adsorbed on the undersurface of PS spheres, and then FeCl_3_ was added to the above solution. After polymerization, a tightly wrapped PPy film on the undersurface of PS spheres was formed, which morphology is shown in Figure 1d. Then, the tightly wrapped PPy film with PS spheres was transferred onto p-TiO_2_. After the PS spheres were peeled off, the PPy nanobowls (b-PPy) were obtained on p-TiO_2_. It can be seen from Figure 1e that the PPy nanobowls uniformly covered the surface in a large area, and a regular 3D structure is formed. Finally, Ag nanoparticles (NPs) were deposited on the surface of b-PPy/p-TiO_2_ to form the Ag/b-PPy/p-TiO_2_ SERS substrate, in which the structure is similar to the bioinspired compound eyes (Figure 1f).

### 3.2. Raman Performance of the Ag/b-PPy/p-TiO_2_ SERS Substrate

**Figure 3:**
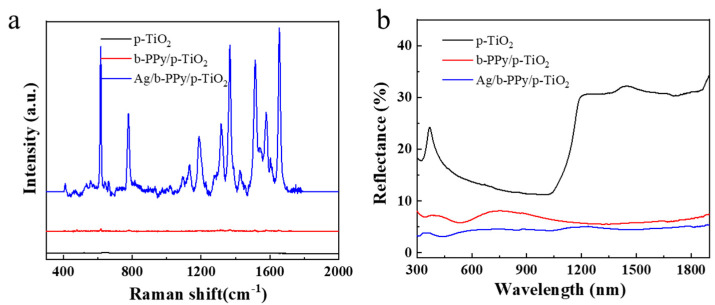
(**a**) SERS spectra of R6G adsorbed on different substrates. (**b**) Reflection spectra of different substrates.

In order to evaluate the advantages of this composite SERS substrate of Ag/b-PPy/p-TiO_2_ on the Raman performance, p-TiO_2_ and b-PPy/p-TiO_2_ were selected as comparison samples. R6G is used as the probe molecule to record the Raman performance on the different substrates. As seen in Figure 3a, there is almost no Raman signal for p-TiO_2_ and b-PPy/p-TiO_2_. However, the Raman signal on the Ag/b-PPy/p-TiO_2_ substrate is significantly higher than that of other SERS substrates. The excellent Raman performance of Ag/b-PPy/p-TiO_2_ can be attributed to the following two aspects. On the one hand, the 3D structure with a rough surface, consisting of the pyramid structure of TiO_2_, the nanobowl structure of PPy, and the uniformly deposited Ag NPs, form a large number of hot spots [28,29,30]. At the same time, the photogenerated electrons generated by the incident light absorbed by PPy are transferred to the Ag nanoparticles, further increasing the electromagnetic field intensity on the silver surface, thus improving the Raman signal of the SERS substrate. Thus, the Raman signal of Ag/b-PPy/p-TiO_2_ is obviously higher than that of others. On the other hand, the 3D bioinspired structure of the Ag/b-PPy/p-TiO_2_ substrate conforms to the equivalent medium theory [31,32], which makes the refractive index from the air to the substrate change gradually, and can improve the absorption of light effectively, as shown in Figure 3b. Moreover, the plasmon resonance generated by Ag NPs can effectively increase light absorption and broaden the spectral absorption range.

**Figure 4:**
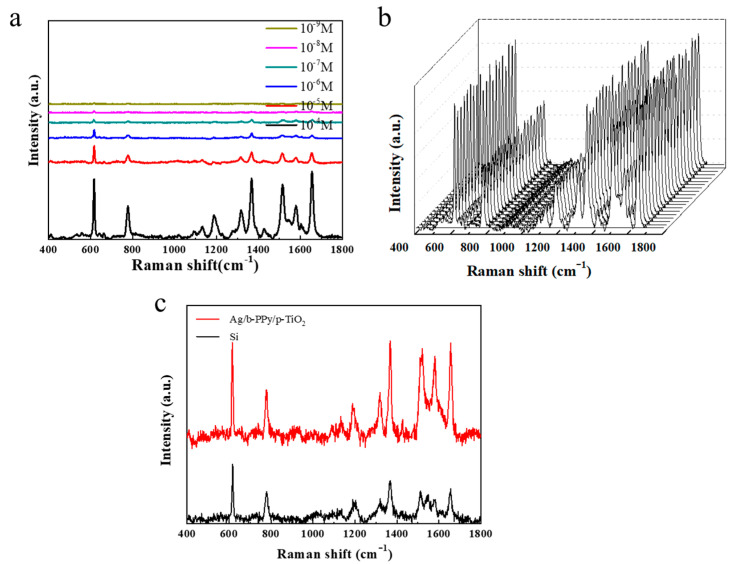
(**a**) Raman spectra of different concentrations of R6G measured on Ag/b-PPy/p-TiO_2_ substrate, the concentrations of R6G is10^−4^, 10^−5^, 10^−6^, 10^−7^, 10^−8^, and 10^−9^ mol/L. (**b**) Raman spectra recorded from 20 random points for 10^−4^ mol/L R6G on the SERS substrate. (**c**) Raman spectra of 10^−2^ mol/L R6G on the Si substrate and 10^−7^ mol/L R6G on the Ag/b-PPy/p-TiO_2_ SERS substrate.

In addition to the high Raman signal intensity, sensitivity, uniformity, and recyclability are also important to measure Raman performance. The lowest detection concentration of R6G is investigated to evaluate the sensitivity of the Ag/b-PPy/p-TiO_2_ substrate. The Raman spectra were collected by immersing the substrate in R6G ethanol solution with concentrations of 10^−4^, 10^−5^, 10^−6^, 10^−7^, 10^−8^, and 10^−9^ mol/L, respectively. It can be seen from Figure 4a that a weak signal can still be detected when the concentration is only 10^−9^ mol/L. This indicates that the Ag/b-PPy/p-TiO_2_ SERS substrate has high sensitivity in Raman detection. Additionally, our Ag/b-PPy/p-TiO_2_ SERS substrate exhibited an impressive SERS performance compared with various other similar reported SERS substrates (Table 1). It can be found from Figure 4b that there is almost no difference in Raman spectra of R6G (10^−4^ mol/L) at 20 random points of Ag/b-PPy/p-TiO_2_. The relative standard deviation (RSD) is about 7%. This is because the Ag/b-PPy/p-TiO_2_ substrate has a highly ordered structure, which will lead to excellent uniformity. The enhancement factor (EF) is a typical parameter to evaluate the enhancement ability of a certain substrate, which can be calculated by referring to the previous works by our research team [20,21]. Here, to obtain EF, 10 μL of 10^−7^ mol/L R6G solution was dropped onto 1 cm^2^ of Ag/b-PPy/p-TiO_2_ substrate and Si wafer, respectively. As shown in Figure 4c, EF = 3.4 × 10^5^ can be calculated based on the peak intensity of 1368 cm^−1^ in the Raman spectra.

### 3.3. Photocatalysis and Recyclability of the Ag/b-PPy/p-TiO_2_ SERS Substrate

**Figure 5:**
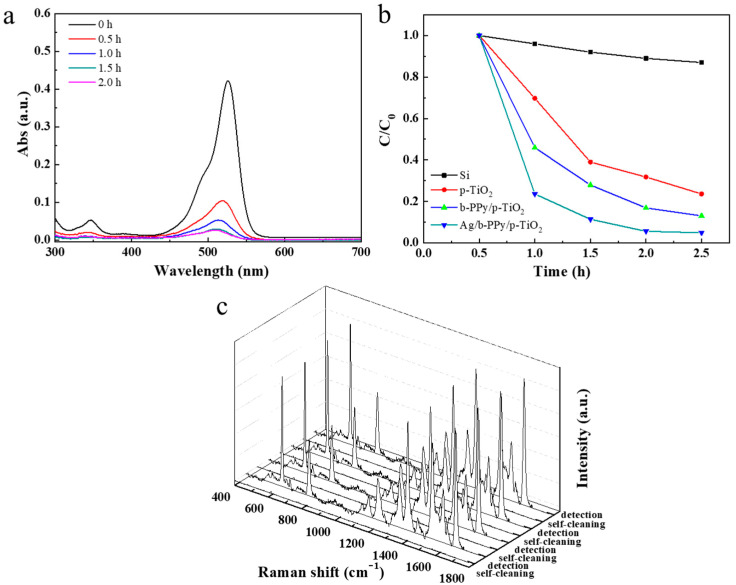
(**a**) UV–vis spectra of R6G under the simulated solar irradiation by the Ag/b-PPy/p-TiO_2_ substrate. (**b**) Photocatalytic activity of different substrates for the degradation of R6G. (**c**) Raman spectra of R6G solution (10^−4^ mol/L) adsorbed on the Ag/b-PPy/p-TiO_2_ SERS substrate before and after UV irradiation for 4 times.

The photocatalytic activity of the Ag/b-PPy/p-TiO_2_ SERS substrate was tested under simulated solar irradiation. Figure 5a shows the influence of Ag/b-PPy/p-TiO_2_ SERS substrate on the degradation of R6G with the change in absorption peak at different times. The maximum absorption peak of 532 nm completely disappeared with the illumination time expanding to 1.5 h. This indicates that R6G is completely degraded. It can be found from Figure 5b that the Ag/b-PPy/p-TiO_2_ SERS substrate has the fastest catalytic degradation rate and the best photocatalytic activity on R6G. This can ensure the self-cleaning ability of the substrate, so as to realize its recyclability. The Raman signal of the Ag/b-PPy/p-TiO_2_ substrate is tested after the adsorption of 10^−4^ mol/L R6G (detection step). When the substrate is treated with the simulated solar irradiation for 2 h, the Raman signal almost completely disappeared (self-cleaning step), as seen in Figure 5c. After four cycles of detection/self-cleaning, the Raman signal intensity of the substrate is almost unchanged, and the RSD with the Raman characteristic peak at 1368 cm^−1^ is 4.1%. These results fully demonstrate the excellent recyclability of the bioinspired Ag/b-PPy/p-TiO_2_ SERS substrate.

**Figure 6:**
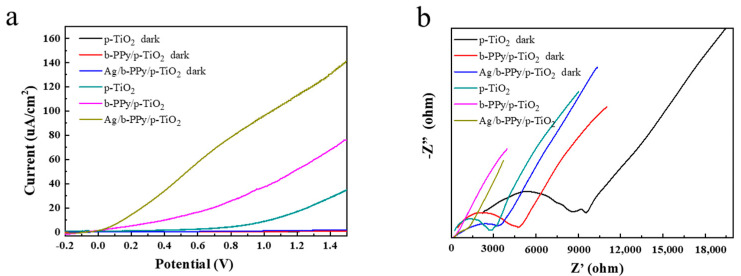
(**a**) Linear sweep voltammograms and (**b**) electrochemical impedance spectra for p-TiO_2_, b-PPy/p-TiO_2_, and Ag/b-PPy/p-TiO_2_.

The main factors affecting photocatalytic activity are the interfacial reaction efficiency, the light utilization efficiency, and the photogenerated charge separation efficiency. The 3D structure with a rough surface facilitates effective liquid/phase catalysis. The multiple bioinspired structure is conducive to effective light utilization. The selected materials for fabricating the substrate conducive to the photogenerated charge separation. To further investigate the reason for the excellent photocatalytic performance of Ag/b-PPy/p-TiO_2_, linear sweep voltammograms (LSVs) and impedance characterization are performed, as shown in Figure 6. Compared with other substrates, Ag/b-PPy/p-TiO_2_ has the highest photocurrent density, as seen in Figure 6a. The reason is that the Schottky barrier formed between Ag NPs and PPy nanobowls makes the photogenerated electrons transfer more easily than the metal NPs and promotes the photogenerated electron–hole separation [38,39]. Meanwhile, TiO_2_ and PPy in these multiple composite materials are an n-type and a p-type semiconductor, respectively. The p–n heterojunction is formed at the interface of the two materials, which can effectively inhibit the photogenerated electron–hole recombination [21,40]. Figure 6b shows that Ag/b-PPy/p-TiO_2_ has a smaller impedance radius. This indicates that Ag/b-PPy/p-TiO_2_ can effectively promote the photogenerated charge carriers transfer, which is more conducive to reducing the electron–hole recombination and improving the photoelectric conversion efficiency.

## 4. Conclusions

In summary, the micropyramid TiO_2_ with antireflection performance was fabricated by soft imprinting, which is simple and easy to operate. After that, PPy nanobowls obtained by self-assembly were covered on the surface of micropyramid TiO_2_, and then Ag NPs were modified on the PPy nanobowls to form Ag/b-PPy/p-TiO_2_ SERS substrate. Finally, the multiple bioinspired composite substrates with good antireflection activity, high photoelectric conversion efficiency, and excellent photocatalysis efficiency were obtained. Due to the high density of hot spots and the ordered structure, the Raman signals with high intensity and high uniformity can be realized. At the same time, the excellent photocatalytic performance enables the recyclability of the bioinspired Ag/b-PPy/p-TiO_2_ SERS substrate.

## Figures and Tables

**Table 1:** Comparison of LOD with similar reported SERS substrate.

SERS Substrate	Conditions	Signal Molecule	LOD	Reference
TiO_2_/ZnO/Ag	Immersion	4-mercaptobenzoic acid	10^−9^ mol/L	[33]
FTO/Ag/ZIF-8	Immersion	4-mercaptobenzoic acid	10^−9^ mol/L	[34]
graphene/Ag/TiO_2_	Dropping	Rhodamine 6G	10^−11^ mol/L	[35]
Ag/air/PPy	-	Rhodamine B	10^−7^ mol/L	[36]
3D TiO_2_-Ag-GO	Dropping	Crystal violet	10^−8^ mol/L	[37]
Ag/b-PPy/p-TiO_2_	Dropping	Rhodamine 6G	10^−9^ mol/L	this work
